# Properties of journal impact in relation to bibliometric research group performance indicators

**DOI:** 10.1007/s11192-012-0747-0

**Published:** 2012-04-25

**Authors:** Anthony F. J. van Raan

**Affiliations:** Centre for Science and Technology Studies, Leiden University, Wassenaarseweg 52, P.O. Box 9555, 2300 RB Leiden, The Netherlands

**Keywords:** Impact factor, Journal impact, Bibliometric analysis, Research group performance

## Abstract

In this paper we present a compilation of journal impact properties in relation to other bibliometric indicators as found in our earlier studies together with new results. We argue that journal impact, even calculated in a sufficiently advanced way, becomes important in evaluation practices based on bibliometric analysis only at an aggregate level. In the relation between average journal impact and actual citation impact of groups, the influence of research performance is substantial. Top-performance as well as lower performance groups publish in more or less the same range of journal impact values, but top-performance groups are, on average, more successful in the entire range of journal impact. We find that for the high field citation-density groups a larger size implies a lower average journal impact. For groups in the low field citation-density regions however a larger size implies a considerably higher average journal impact. Finally, we found that top-performance groups have relatively less self-citations than the lower performance groups and this fraction is decreasing with journal impact.

## Introduction

The discussion on the meaning of journal impact for both the assessment of the standing of journals as well as its use in evaluation practices regularly flares up. A striking example is the discussion in *Nature* (initiated by the paper of Lawrence 2003) in which researchers, referring to the work of Seglen (1992, 1994), fulminate against the supposed dominant role of journal status and journal impact factors in the present-day life of a scientist. The most important finding of Seglen was the poor correlation between the impact of publications and journal impact at *the level of individual publications*. Seglen therefore concluded that the use of journal impact as an indicator for research performance evaluation is inappropriate. We stress that even when peer-review based assessment of journal status would completely replace citation-based (bibliometric) measures of journal impact, the above discussed poor correlation would remain. This is due to the skew distribution of citations over individual publications in any journal and even in any entity, regardless of whatever peer-review based journal standing indicator.

However, Seglen also found that *aggregating publications* in classes of journal impact yielded a high correlation between the average number of citations per publication and journal impact. We found (van Raan 2006a) that aggregating publications in a more institutional way, namely the publications of research groups, also shows a significant correlation between the average number of citations per publication of research groups, and the average journal impact of these groups.

In Seglen’s work the calculation of journal impact was based on the ISI^1^ journal impact factor. This measure has important disadvantages for bibliometric studies (Moed and Van Leeuwen 1995, 1996; Vanclay 2011). In our work we use the more sophisticated journal impact indicators developed with a long standing experience by our institute (CWTS). We discussed in recent papers statistical properties of the relations between journal impact and other bibliometric indicators (van Raan 2006a, b, 2008a, b). Together with further results we present in this paper a compilation of the findings of the above papers in order to have a concise overview of the most relevant properties of journal impact. In this paper we argue that journal impact, even calculated in a sufficiently advanced way, becomes important in evaluation practices based on bibliometric analysis only at an aggregate level.

As described in our earlier studies we use a large data set covering all university chemistry groups in the Netherlands, covering in the 10-year period 1991–2000 in total 157 research groups, about 700 senior researchers with about 18,000 publications (WoS) and 175,000 citations (excluding self-citations) to these publications. For a detailed discussion of the data material we refer to the above mentioned work and for the calculation of the indicators we refer to our earlier work (van Raan 1996, 2004). The indicators are the standard CWTS bibliometric indicators explained in the text box here below.^2^

**Table:** CWTS standard bibliometric indicators

Number of publications (***P***) in WoS-covered journals of a specific entity in a given time period
Number of citations without self-citations (***C***) received by ***P*** during the entire period
Average number of citations per publication without self-citations (***CPP***)
Percentage of publications not cited (in the given time period), ***Pnc***
Average journal impact for each journal used by the specific entity (***JCS***, journal citation score), without self-citations; as almost always a set of journals is used, we calculate the weighted average ***JCSm***; for the calculation of ***JCSm*** the same publication and citation counting procedure, time windows, and article types are used as in the case of ***CPP***
Taking all journals of a field, we calculate the average field-based impact as an international reference level for the specific entity (***FCS***, field citation score), without self-citations. In the case of more than one field (as almost always) we use the weighted average ***FCSm***; for the calculation of ***FCSm*** the same publication and citation counting procedure, time windows, and article types are used as in the case of ***CPP***
Comparison of the actually received impact of the specific entity with the world-wide average based on ***JCSm*** as a standard, without self-citations, indicator ***CPP***/***JCSm***
Comparison of the actually received impact of the specific entity with the world-wide average based on ***FCSm*** as a standard, without self-citations, indicator ***CPP***/***FCSm***
Ratio ***JCSm***/***FCSm*** indicating whether the impact of a journal is above (ratio >1) or below (ratio <1) the field average
Percentage of self-citations ***sc***

The structure of our paper is as follows. We present in the next section results on four main topics: the distribution of journal impact over individual publications (the lowest aggregation level) and over research groups (the next higher aggregation level); the relation between journal impact and actual citation impact of groups and the influence of research performance; the group-size dependence of journal impact in relation to field-specific citation density; and the relation between journal impact and self-citation. In the last section we summarize our main conclusions.

## Results

### Distribution of journal impact for individual publications and for groups

One of the crucial characteristics of a publication is the journal in which it appears. We calculated for each of the individual publications in our data set its ***JCS*** value. This is the average number of citations per publication of the journal in which the publication was published, with the same time window for citation counting as used for counting of the citations to the publication, and taking into account the type of publication (normal paper, letter, review). So in the case of a review paper, the corresponding ***JCS*** is calculated only with the reviews in the journal. Thus, the important differences of our journal indicator ***JCS*** with the ISI journal impact factor are:

- ***JCS*** values are calculated with the same citation window as used for the citation counting of the publication; this window is at least 4 years (for the ISI impact factor the window is only 2 years);
- If the publication is a review, a letter of a normal paper, the ***JCS*** values are calculated for only review, letters or normal papers in the journal, thus our ***JCS*** takes into account document type.

**Fig. 1:**
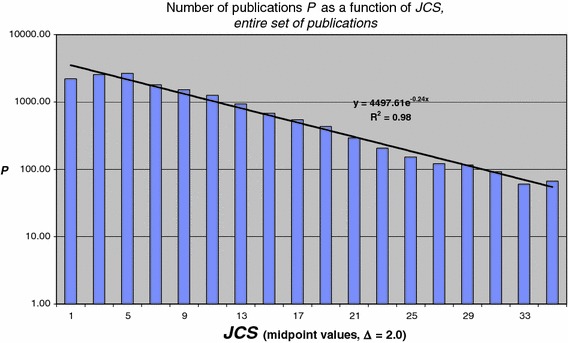
Distribution function ***P***(***JCS***) for all publications (with more than zero citations) in the entire set (class width Δ ***JCS*** = 2.0)

We calculated the distribution of journal impact ***JCS*** for our entire set of publications, i.e., the number of publications ***P***(***JCS***) as a function of ***JCS*** values. Only a very small part of the entire publication population belongs to the very high-value ***JCS*** classes (i.e., ***JCS*** > 30), making the distribution for this high ***JCS*** part very noisy (see van Raan 2006a). If we restrict the analysis to the publications with values of ***JCS*** ≤ 35, we cover 97 % of the publications. The distribution function for these publications is shown in Fig. 1. We find an exponential relation given by^3^

$$ P\left( {JCS} \right) = \beta \,{ \exp }\left[ { - 0. 2 4\left( {JCS} \right)} \right] $$

The above observation supports our earlier theoretical work (van Raan 2001a, b) on an ab initio model to explain the distribution of journal impact in a large set of publications.

**Fig. 2:**
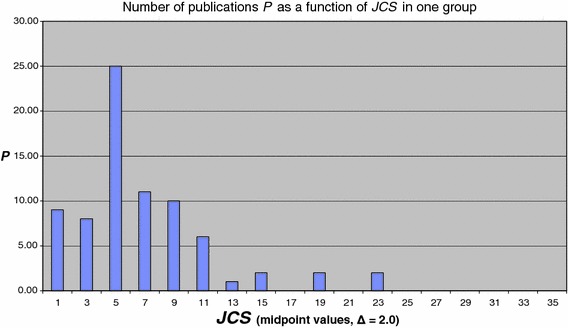
Distribution function ***P***(***JCS***) for the publications (with more than zero citations) of one group (class width Δ ***JCS*** = 2.0)

Next to the distribution of journal impact for the entire set of publications, it is interesting to see how this distribution looks for just one research group. We show an example in Fig. 2. Research groups cover subsets of the entire set (on average around 100 publications) and thus a *group average* ***JCSm*** can be calculated, based on around 10 journals used by the group. The figure shows that indeed even within one group there is quite a large range of journal impact. But we also observe that most of the work is published in a small group of journals. Typically, 50 % or more of the publications of a group are published in 2 to 3 journals. In our example most of the publications are published in journals with a ***JCSm*** value around 5.0. These journals have the largest influence on the ***JCSm*** value of the group.

**Fig. 3:**
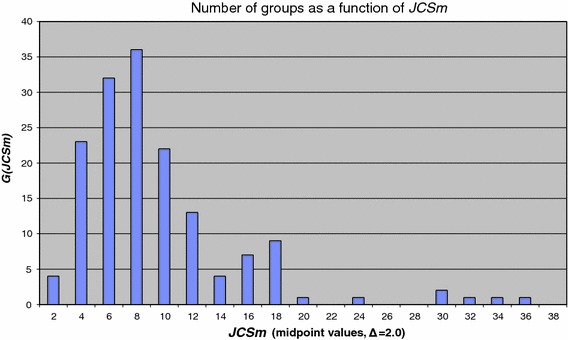
Distribution function ***G***(***JCSm***) (class width Δ ***JCSm*** = 2.0)

Now we move from the lowest aggregation level, individual publications, to the next aggregation level, research groups. In Fig. 3 we present the distribution of the group average journal impact ***JCSm*** over research groups, i.e., ***G***(***JCSm***), the number of chemistry groups as a function of ***JCSm*** values.

**Fig. 4:**
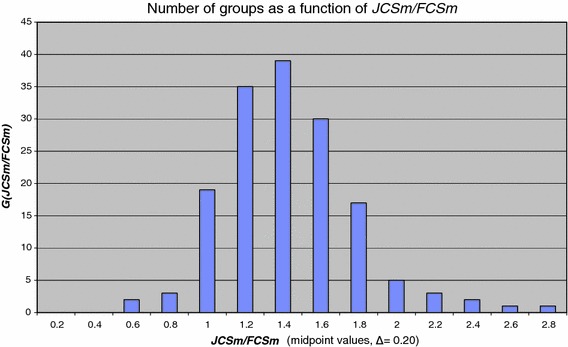
Distribution function ***G***(***JCSm***/***FCSm***) (class width Δ ***JCSm***/***FCSm*** = 0.20)

Because journal impact may differ strongly among fields of science, the field-normalized journal impact indicator ***JCSm***/***FCSm*** is a more appropriate measure to assess for instance whether a group is publishing in the better journals of the field, in that case ***JCSm***/***FCSm*** > 1. In Fig. 4 we present the distribution of ***JCSm***/***FCSm*** over research groups, i.e., ***G***(***JCSm***/***FCSm***), the number of chemistry groups as a function of ***JCSm***/***FCSm*** values.

We clearly see that the distribution function changes from skew to almost normal if group average journal impact factors are field-normalized.

### Journal impact and actual citation impact of groups

**Fig. 5:**
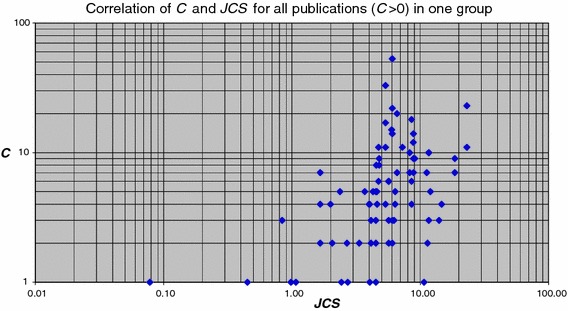
Correlation of the number of citations (***C***) received by the individual publications of one group with the ***JCS*** values of the journal in which the publication was published

How is the number of citations received by individual publications related to the journal impact? In Fig. 5 we show the correlation of the number of received citations with the ***JCS*** of the journal in which the publication was published. As each point in the graph is an individual publication, ***P*** = 1 and therefore in this case ***C*** is equal to ***CPP***. Similar to the findings of Seglen, there is no significant relation between both indicators. Obviously the journal impact is an inappropriate predictor of the impact of an individual publication.

**Fig. 6:**
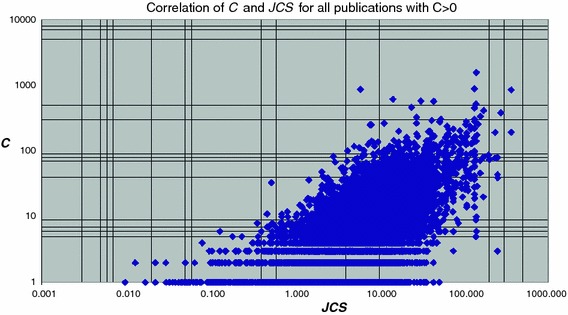
Correlation of the number of citations (***C***) received by the individual publications of the entire set (i.e., all groups together) with the ***JCS*** values of the journal in which the publication was published

In Fig. 6 we present a similar analysis, but now for all publications in the entire set, i.e., all 157 groups together. Now a slight relation between ‘citedness’ of individual publications and journal impact is visible. But again it is clear that for an individual publication the ***JCS*** value is not a proper indicator of impact.

**Fig. 7:**
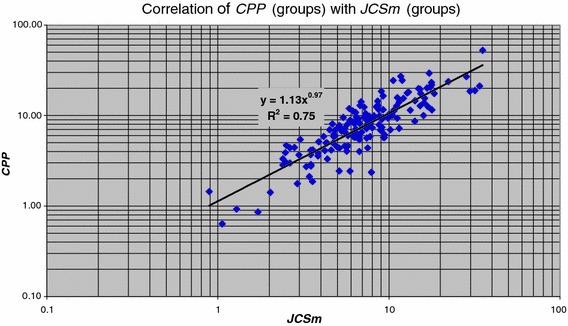
Correlation of ***CPP*** with the ***JCSm*** values for all groups

Next we move from the lowest aggregation level, individual publications, to one aggregation level higher, research groups. Instead of ***C*** (which is the same as ***CPP*** for an individual publication) we have ***CPP***, and instead of ***JCS*** we now have ***JCSm***. The relation between ***CPP*** of a group with the ***JCSm*** of a group is presented in Fig. 7 for all research groups. We find for the whole set of research groups

$$ {\user2{CPP}} = 1.13\,{\user2{JCSm}}^{0.97}$$

which means that ***CPP*** *at the aggregation level of a research group* is related in a very simple, almost proportional manner to ***JCSm***.

Thus, by the ‘phase transition’ to a higher aggregation level a significant relation is found between average impact of the publications of a group (the actual impact) and the average impact of journals of a group.

**Fig. 8:**
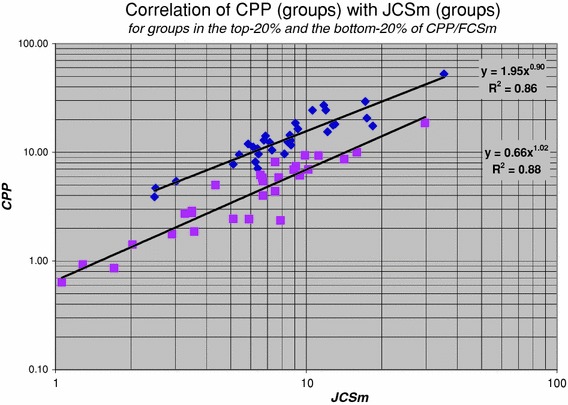
Correlation of ***CPP*** with the ***JCSm*** values for the top-20 % (of ***CPP***/***FCSm***) groups (indicated with *diamonds*), and for the bottom-20 % groups (indicated with *squares*)

By dividing the authors into a highly cited group and a less cited group, Seglen concluded that the highly cited authors tend to publish somewhat more in journals with a higher impact than the less cited authors. Yet this difference was insufficient to explain the difference in impact between the two groups. According to Seglen, on average highly cited authors are in all journal impact classes more successful. To investigate this phenomenon on the level of research groups, we make a distinction between top- and lower performance groups on the basis of our field-specific impact indicator ***CPP***/***FCSm***. Thus, we calculate a similar correlation as shown in Fig. 7, but now we restrict the analysis to the research groups in the top-20 % and in the bottom-20 % of the ***CPP***/***FCSm*** distribution. The results are presented in Fig. 8. We clearly notice the differences and similarities between the two subsets. Both the top-20 % as well as the bottom-20 % groups generally have more citations per publication (***CPP***) as a function of journal impact (***JCSm***). Clearly, the top-20 % groups generally have higher ***CPP*** values. Remarkably, the top-20 % as well as the bottom-20 % groups publish in more or less the same range of journal impact values.

Thus, the important observation is that top-performance groups are, on average, more successful in the *entire range* of journal impact. In other words, they perform better in the lower-impact journals as well as in the higher-impact journals. This nicely confirms Seglen’s findings as discussed above. However, we also observe that the top-20 % groups have a slight preference for the higher-impact journals. Another interesting finding is the difference in power law behaviour between the top-20 % and the bottom-20 % groups: ***CPP*** increases with ***JCSm*** for the top-20 % groups somewhat less stronger (exponent 0.90) than for the bottom-20 % groups (exponent 1.02).^4^

The coefficients of the power law equations provide a quantitative measure of the extent to which the top-20 % groups have a higher average number of citations per publication (***CPP***) for the *same* journal impact (***JCS***) values as compared to lower performance (bottom-20 %) groups. The ratio of the coefficients is 2.95. Thus, the top-groups perform in terms of citations per publications (***CPP***), a factor of about 3 better than the lower performance groups *in the same journals.* 5 Also this finding is in agreement with Seglen’s work, he finds a factor between 1.5 and 3.5.

**Fig. 9:**
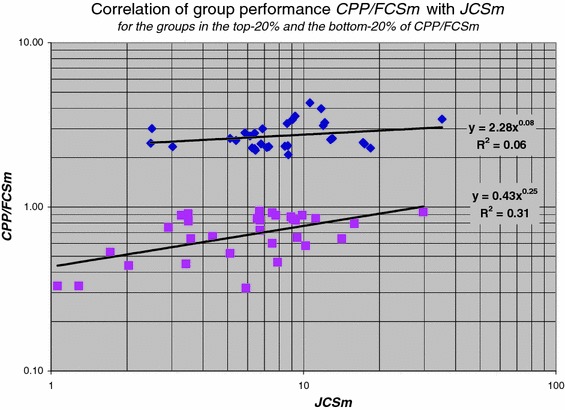
Correlation of ***CPP***/***FCSm*** with the ***JCSm*** values for the top-20 % (of ***CPP***/***FCSm***) groups (indicated with *diamonds*), and for the bottom-20 % groups (indicated with *squares*)

Another way of looking at the relation between group performance and the average journal impact of the group is shown in Fig. 9. We see that for the top-20 % groups there is no difference in performance between groups publishing in the lower-impact journals and the groups publishing in the higher-impact journals. Applied research generally has lower-impact journals as compared to basic research and thus our results nicely show that this does not influence performance measured by our ***CPP***/***FCSm*** indicator. In other words, top-groups can be identified in applied research as well as in basic research. But we also see that the top-20 % groups do not publish in the journals with the lowest impact.

For the bottom-20 % groups we observe that the better performing groups tend to publish in the higher-impact journals, but the significance of this relation is low.

### Size dependence of journal impact in relation to field citation density

**Fig. 10:**
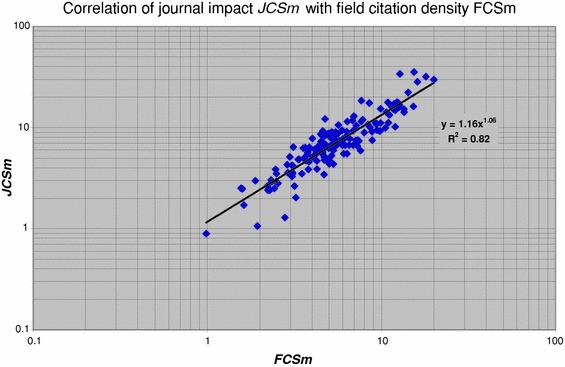
Correlation of ***JCSm*** with ***FCSm*** for all groups

**Fig. 11:**
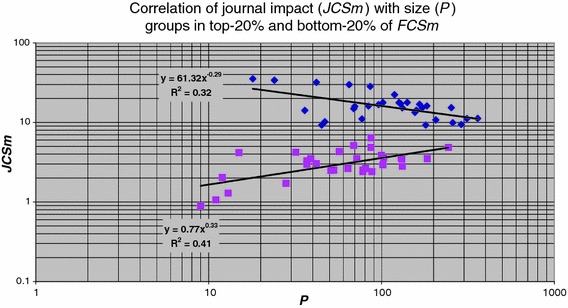
Correlation of journal impact (***JCSm***) with size (***P***) for groups in fields with a high (*diamonds*) and a low (*squares*) citation density (***FCSm***)

Generally there is a strong correlation between the average journal impact of a group (***JCSm***) and the citation density of the fields in which the group is active (***FCSm***), see Fig. 10. This is to be expected as a field is defined as a specific set of journals (i.e., a WoS journal category). However, when looking in more detail, we find interesting properties of the journal impact of groups in relation to the average field citation density. So far we did not take into account the size of a group, measured by the number of publications (***P***). But in our earlier work (van Raan 2008a) we showed that size plays an important role. We illustrate this with Fig. 11.

Here we make a distinction between research groups active in fields that belong to the top-25 % of the field citation density (***FCSm***)—in most cases these are the more basic research oriented fields and those groups active in fields with the lowest 25 % of the field citation density—mostly the more applied research oriented fields. We stress that this division does not imply any difference in performance, as explained earlier. For both subsets we calculated the ***JCSm*** values as a function of size. We find that for the *high* field citation-density groups a larger size implies a *lower* average ***JCSm*** value. This implies for research groups operating in high field citation-density regions that a larger number of publications will lead to a somewhat lower average journal citation impact. Thus, ‘expanding in size’ may take place within the same field citation-density region, but it will generally include publications in journals with a lower impact.

In contrast, for groups in the *low* field citation-density regions a larger size implies a considerably higher average ***JCSm*** value. Thus, for groups operating in low field citation-density regions a larger number of publications can be seen as an ‘expansion’ to journals with a higher impact. In our previous work (van Raan 2008a) we presented a ‘landscape’ model to explain these observations and their quantitative properties.

### Journal impact and self-citation

**Fig. 12:**
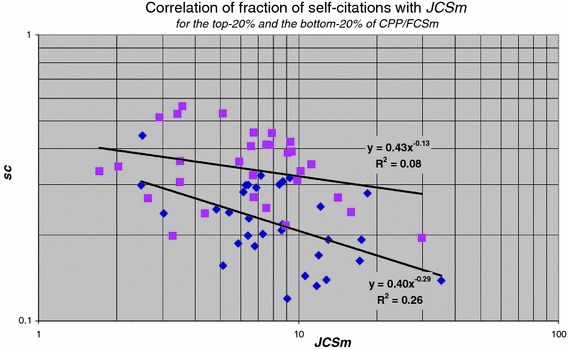
Correlation of the fraction of self-citations (***sc***) with average journal impact (***JCSm***) for the higher (*diamonds*) and the lower performance (*squares*) research groups, top-20 % and bottom-20 % of the ***CPP***/***FCSm*** distribution, respectively

Self-citation is a well know phenomenon in science (Aksnes 2003; Fowler and Aksnes 2007; Glänzel et al. 2004, 2006; Glänzel and Thijs 2004a, b; Thijs and Glänzel 2006). We found (van Raan 2008b) that the fraction of self-citations tends to decrease with journal impact. If we select the top-20 % and the bottom-20 % of the ***CPP***/***FCSm*** distribution we observe that the top-performance groups (top-20 % of the ***CPP***/***FCSm*** distribution) have relatively less self-citations than the lower performance groups (bottom-20 % of the ***CPP***/***FCSm*** distribution), and this fraction is also decreasing more rapidly with journal impact, see Fig. 12.

We conclude that the fraction of self-citations tend to decrease with journal impact and with performance. The significance however is not very high.

## Summary of the main conclusions

We argued that the ISI journal impact factor is unsuitable for the use in bibliometric studies in general and particularly for evaluation studies. The journal impact indicator developed by the CWTS has considerably better properties. We showed that there is a remarkable ‘phase transition’ in the meaning of journal impact when going from the lowest aggregation level—individual publications—to a higher aggregation level—research groups, about two orders of magnitude larger. For individual publications even a more sophisticated journal impact is still an inappropriate measure predictor of the actual impact of a publication, whereas for research group the average journal impact correlates well with the actual impact of a group.

The distribution of journal impact over individual publications follows an exponential function with high significance and this is a nice example that not all relations of bibliometric measures follow power laws. In the relation between average journal impact and actual citation impact of groups, the influence of research performance is substantial. Top-performance as well as lower performance groups publish in more or less the same range of journal impact values, but top-performance groups are, on average, more successful in the entire range of journal impact. In other words, top-groups perform better in the lower impact as well as in the higher-impact journals: in terms of citations per publications they perform a factor 3 better than the lower performance groups in the same journals. We also observe that the top-20 % groups have a slight preference for the higher-impact journals and they do not publish in the journals with the lowest impact. The average number of citations increases with average journal impact for the top-20 % groups somewhat less strong than for the bottom-20 % groups.

We find that for the high field citation-density groups a larger size implies a lower average journal impact: for research groups operating in high field citation-density regions a larger number of publications will lead to a somewhat lower average journal citation impact. For groups in the low field citation-density regions however a larger size implies a considerably higher average journal impact. Finally, we found that top-performance groups have relatively less self-citations than the lower performance groups and this fraction is decreasing with journal impact.

As long as they exist, journals do and will play an important role in the assessment of the quality of research, regardless whether the assessment is based on peer review only, on bibliometric analysis, or a combination of both. Therefore it is of crucial importance to know the properties of journal impact in relation to other bibliometric indicators. Hopefully, the results reported in this paper will stimulate the careful use of journal impact measures.

## Footnotes

1 The former Institute for Scientific Information (ISI) in Philadelphia was the producer of the Science Citation Index and related indexes. Now Thomson Scientific is the producer of the citation indexes combined in the Web of Science (WoS) data system.

2 Recently, we adopted a new system of bibliometric indicators, see Waltman et al. (2011a, b). For the results presented in this paper, this change of indicator system is not relevant.

3
**β** is a constant factor which can be determined empirically from the plot (**β** = 4,497.61).

4 By randomly removing 10 groups in the set of research groups and recalculating the correlation functions, we estimate that the uncertainty in the power law exponents is about ±0.04.

5 For top-10% and bottom-10% the ratio is 3.45, for top-50% and bottom-50% we find 1.87.
